# Upconversion-based nanosystems for fluorescence sensing of pH and H_2_O_2_†

**DOI:** 10.1039/d0na01045f

**Published:** 2021-03-18

**Authors:** Chunning Sun, Michael Gradzielski

**Affiliations:** Stranski-Laboratorium für Physikalische und Theoretische Chemie, Institut für Chemie, Technische Universität Berlin Strasse des 17. Juni 124 10623 Berlin Germany chunning.sun@campus.tu-berlin.de michael.gradzielski@tu-berlin.de

## Abstract

Hydrogen peroxide (H_2_O_2_), a key reactive oxygen species, plays an important role in living organisms, industrial and environmental fields. Here, a non-contact upconversion nanosystem based on the excitation energy attenuation (EEA) effect and a conventional upconversion nanosystem based on the joint effect of EEA and fluorescence resonance energy transfer (FRET) are designed for the fluorescence sensing of H_2_O_2_. We show that the upconversion luminescence (UCL) is quenched by MoO_3−*x*_ nanosheets (NSs) in both systems due to the strong absorbance of MoO_3−*x*_ NSs in the visible and near-infrared regions. The recovery in UCL emissions upon addition of H_2_O_2_ enables quantitative monitoring of H_2_O_2_. Benefiting from the non-contact method, hydrophobic OA-NaYF_4_:Yb,Er can be used as the luminophore directly and ultrahigh quenching efficiency (99.8%) is obtained. Moreover, the non-contact method exhibits high sensitivity toward H_2_O_2_ with a detection limit of 0.63 μM, which is lower than that determined by simple spectrophotometry (0.75 μM) and conventional upconversion-based nanocomposites (9.61 μM). As an added benefit, the same strategy can be applied to the sensing of pH, showing a broad pH-responsive property over a range of 2.6 to 8.2. The successful preparation of different upconversion-based nanosystems for H_2_O_2_ sensing using the same material as the quencher provides a new design strategy for fluorescence sensing of other analytes.

## Abstract 2

Non-contact and conventional upconversion-based nanosystems were designed for the fluorescence sensing of pH and H_2_O_2_.

## Graphical abstract

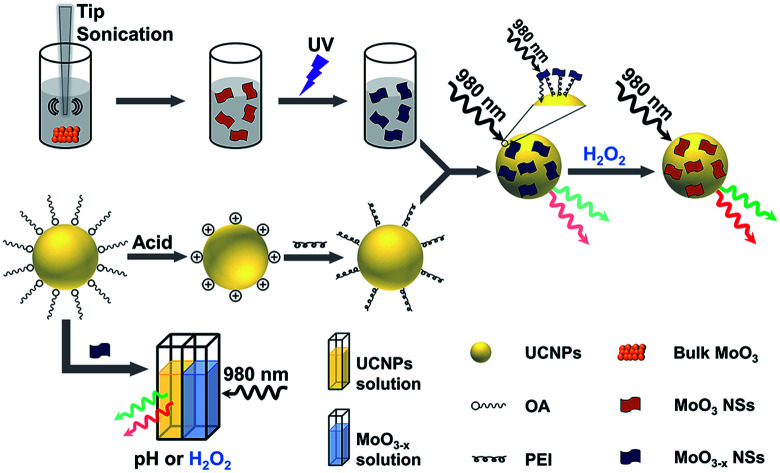

## Introduction

Hydrogen peroxide (H_2_O_2_), an important bioactive molecule in living systems, plays an essential role in the physiological process including signal transduction, cell proliferation, differentiation, and maintenance.^1,2^ Abnormal production or accumulation of H_2_O_2_ will lead to severe damage to DNA and proteins, causing a series of serious diseases,^3–7^ such as diabetes, Alzheimer's and Parkinson's disease, cardiovascular disorders, and even cancer. Additionally, H_2_O_2_ is widely used as a bleaching agent and sterilant in industrial and environmental fields,^8,9^ such as food processing, drinking water treatment, packaging, and organic pollutant degradation. However, exposure to high concentrations of H_2_O_2_ is a great threat to organisms.^10,11^ Therefore, quantitative detection of H_2_O_2_ is of great importance for monitoring its potential risk.

Optical methods *via* fluorescence changes have attracted considerable attention, as the fluorometric approach is a non-destructive method that can be simply and rapidly performed with high sensitivity and selectivity.^12^ In contrast to conventional fluorescence probes (such as organic dyes, carbon nanomaterials, and semiconductor quantum dots), upconversion nanoparticles featuring large anti-Stokes shifts, excellent chemical- and photo-stability, sharp multicolor emissions, and low toxicity have been regarded as a promising class of luminophores.^13^

Up to now, a variety of functional materials including organic dyes,^14–16^ noble metals,^17–19^ quantum dots,^20–22^ carbon nanomaterials,^23–25^ and two-dimensional materials^26–28^ has been employed to couple with upconversion nanoparticles to construct fluorescence probes, realizing quantitative detection of inorganic ions,^29–31^ pH,^32–34^ small molecules,^35–38^ and nucleic acids.^39–42^ Most of the upconversion-based probes rely on the fluorescence resonance energy transfer (FRET) process, in which a very short distance between the upconversion nanoparticles and absorbers is required. Moreover, in order to obtain high-sensitivity detection, high-quality upconversion nanoparticles with strong emission and high upconversion efficiency are employed, which are commonly prepared by applying oleic acid (OA) as the ligand. The oleate-capped upconversion nanoparticles are hydrophobic and prone to disperse in nonpolar solvents, whereas hydrophilic upconversion nanoparticles are required for typical sensing applications of interest. Therefore, the hydrophobic-to-hydrophilic transition of upconversion nanoparticles is essential.^43^

Herein, we propose different upconversion nanosystems for H_2_O_2_ sensing using MoO_3−*x*_ nanosheets (NSs) as the energy acceptor based on either the excitation energy attenuation (EEA) effect or the joint effect of the EEA and FRET, owing to the strong absorbance of MoO_3−*x*_ NSs in both visible and near-infrared (NIR) regions. By coupling of MoO_3−*x*_ NSs solution and oleate-capped NaYF_4_:Yb,Er upconversion nanoparticles (abbreviated as OA-UCNPs) solution, a EEA-based upconversion nanosystem for sensing of H_2_O_2_ in the non-contact mode is designed, where MoO_3−*x*_ NSs act as the energy acceptor of the incident light for the activation of UCNPs. Additionally, this system can be used for pH sensing as well. Benefiting from the non-contact method, hydrophobic OA-UCNPs can be used directly for the sensing and ultrahigh quenching efficiency (99.8%) can be reached. Meanwhile, by the integration of hydrophilic UCNPs and MoO_3−*x*_ NSs, we are able to prepare conventional upconversion-based nanocomposites for H_2_O_2_ sensing *via* the joint effect of the EEA and FRET, where MoO_3−*x*_ NSs act as the energy acceptor of not only the 980 nm exciting light for UCNPs but also fluorescence emissions of UCNPs. To the best of our knowledge, this is the first upconversion-based nanoprobe for the sensing of one analyte by two different systems while using the same material as an energy acceptor.

## Experimental section

### Materials

Yttrium(iii) acetate tetrahydrate (99.9%), ytterbium(iii) acetate hydrate (99.9%), erbium(iii) acetate hydrate (99.9%), MoO_3_ (99.95%) were purchased from Alfa Aesar, 1-octadecene (ODE, 90%), oleic acid (OA, 90%), sodium hydroxide (NaOH, ≥98%), ammonium fluoride (NH_4_F, ≥98%), methanol (99.8%), cyclohexane (99.5%), ethanol (≥99.8%), formic acid (≥98%), polyethylenimine (PEI, branched, *M*_w_ ∼25 000) were obtained from Sigma-Aldrich. Milli-Q water (18.2 MΩ cm at 25 °C) was used in all experiments.

### Characterization

Fourier transform infrared (FT-IR) spectra were recorded in transmission mode on a Thermo Scientific Nicolet iS5 FT-IR spectrometer with the KBr method. X-ray photoelectron spectroscopy (XPS) was measured with a Thermo Fisher Scientific ESCALAB 250Xi instrument. Transmission electron microscopy (TEM) and energy-dispersive X-ray spectroscopy (EDS) were performed on the FEI Tecnai G2 20 S-TWIN with a LaB_6_ cathode operated at 200 kV. UV-vis absorption spectra were acquired on a CARY 50 spectrophotometer. Powder X-ray diffraction (XRD) measurements were performed on a Philips X'Pert MPD Pro X-ray diffractometer at a scanning rate of 4° min^−1^ in the 2*θ* range from 10° to 80° (Cu Kα radiation, *λ* = 0.15406 nm). *ζ*-Potential measurements were carried out on an Anton Paar Litesizer™ 500 instrument. Upconversion luminescence (UCL) emission spectra were obtained on a fiber-coupled spectrometer (Ocean HDX, Ocean Optics) with an external 980 nm continuous-wave (CW) laser (0–5 W, Roithner Lasertechnik GmbH) at room temperature (RT). Quartz cuvettes (0.7 mL, 10 mm × 2 mm light path) were used for UV-vis absorption and UCL measurements.

### Synthesis of MoO_3−*x*_ NSs

MoO_3−*x*_ NSs were prepared according to the previous publication with minor modifications.^44,45^ In a typical process, 1.5 g bulk MoO_3_ powder was ground with 0.3 mL of acetonitrile for 30 min and then added to a water/ethanol solution (25 mL, v/v = 1/1). The dispersion was then probe-sonicated for 2 h at 100 W (Branson Digital Sonifier W-250D) at a 5 s ON and 2 s OFF pulse. To avoid overheating of the solvent, the beaker filled with MoO_3_ dispersion was immersed in an ice bath during sonication. The light blue supernatant containing a high concentration of MoO_3_ NSs (denoted as S-MoO_3_ NSs) was collected *via* centrifugation at 7000*g* for 30 min. For the preparation of MoO_3−*x*_ NSs, the supernatant dispersion was filled into a quartz glass vial and irradiated with a UV lamp (254 & 365 nm, 15 W) for 5 h, dark blue MoO_3−*x*_ NSs solution was finally obtained, and the MoO_3−*x*_ NSs solution was then diluted to 2 mg mL^−1^ by water and ethanol (v/v = 1/1) solution, and stored at 4 °C for further use.

### Synthesis of OA-UCNPs

As previously reported, the synthesis of oleate-capped NaYF_4_: 20 mol% Yb, 2 mol% Er was carried out by employing OA as ligand *via* a high-temperature coprecipitation method.^46^ Briefly, in a 100 mL round flask, 3.12 mL of Y(CH_3_COO)_3_ (0.2 M), 0.8 mL of Yb(CH_3_COO)_3_ (0.2 M) and 0.8 of mL Er(CH_3_COO)_3_ (0.02 M) were mixed with 6 mL of OA and 14 mL of ODE at RT. The mixture solution was first heated to 110 °C for 30 min to evaporate the water and then heated to 160 °C for 40 min to form lanthanide-oleate complexes, followed by cooling down to 50 °C. A methanolic solution (10 mL) containing 3.2 mmol of NH_4_F and 2.0 mmol of NaOH was slowly added and then stirred at 50 °C for 30 min. After evaporating the methanol, the solution was heated to 310 °C at a rate of 10 °C min^−1^ and maintained for 30 min under nitrogen atmosphere. After cooling down to RT, OA-UCNPs were precipitated out with the addition of excess ethanol, collected after washing three times with the ethanol, and finally dissolved in cyclohexane for further use.

### Preparation of ligand-free UCNPs

Ligand-free UCNPs were prepared using our previously reported method.^47^ 5 mmol of formic acid was directly added to 2 mL of cyclohexane solution containing 20 mg of OA-UCNPs, ligand-free UCNPs were precipitated out after shaking for 10 s at 3000 rpm on a vortex mixer. Bare UCNPs were obtained after centrifugation and washing once with ethanol and three times with water and finally dissolved in water.

### Synthesis of UCNPs/MoO_3−*x*_ nanocomposites

To synthesize UCNPs/MoO_3−*x*_ nanocomposites, PEI-capped UCNPs (abbreviated as PEI-UCNPs) was first prepared. Typically, 4 mL ligand-free UCNPs solution (5 mg mL^−1^) were added to a vial containing 4 mL PEI solution (10 mg mL^−1^), followed by overnight stirring. PEI-UCNPs were collected after centrifugation at 16 000*g* for 30 min and washing three times with water, and finally dispersed in water with a concentration of 1 mg mL^−1^. UCNPs/MoO_3−*x*_ nanocomposites were prepared by mixing 0.5 mL PEI-UCNPs solution with an appropriate amount of MoO_3−*x*_ NSs solution, the mixture was first shaken for 3 min (3000 rpm) on a vortex mixer and then ultrasonicated for 5 min. UCNPs/MoO_3−*x*_ nanocomposites were then collected by centrifugation at 7000*g* for 30 min, washed three times with water, and redispersed in water.

### Non-contact fluorescence sensing of pH

To detect pH in the non-contact mode, OA-UCNPs dispersed in cyclohexane with a concentration of 1 mg mL^−1^ were sealed in a quartz cuvette, the cuvette was then aligned with the other cuvette containing 1 mg mL^−1^ MoO_3−*x*_ NSs solution with different pH. The pH was adjusted by either 50 mM NaOH or 50 mM HCl ethanol/H_2_O (v/v = 1/1) solution. The cuvette containing MoO_3−*x*_ NSs was put in front of the other one containing OA-UCNPs solution, and the UCL spectra were collected under the excitation of a 4 W 980 nm CW laser.

### Non-contact fluorescence sensing of H_2_O_2_

The non-contact sensing procedure for the H_2_O_2_ was similar to that of the non-contact pH sensing, except that MoO_3−*x*_ NSs were dissolved in acetate buffer (50 mM, pH 4.5, ethanol/H_2_O, v/v = 1/1) with different concentrations of H_2_O_2_.

### Fluorescence sensing of H_2_O_2_ by UCNPs/MoO_3−*x*_ nanoassemblies

To detect H_2_O_2_, 0.5 mg mL^−1^ of UCNPs/MoO_3−*x*_ aqueous solution (0.35 mg mL^−1^ MoO_3−*x*_ NSs) and different concentrations of H_2_O_2_ (0.4 mL) were added to 0.1 mL acetate buffer (50 mM, pH 4.5, DMF/H_2_O, v/v = 1/1), The mixture was then incubated at RT for 2 h, and the UCL spectra were measured under the excitation of a 4 W 980 nm CW laser.

## Results and discussion

### Design principle of upconversion-based nanosystems for H_2_O_2_ and pH

**Fig. 1:**
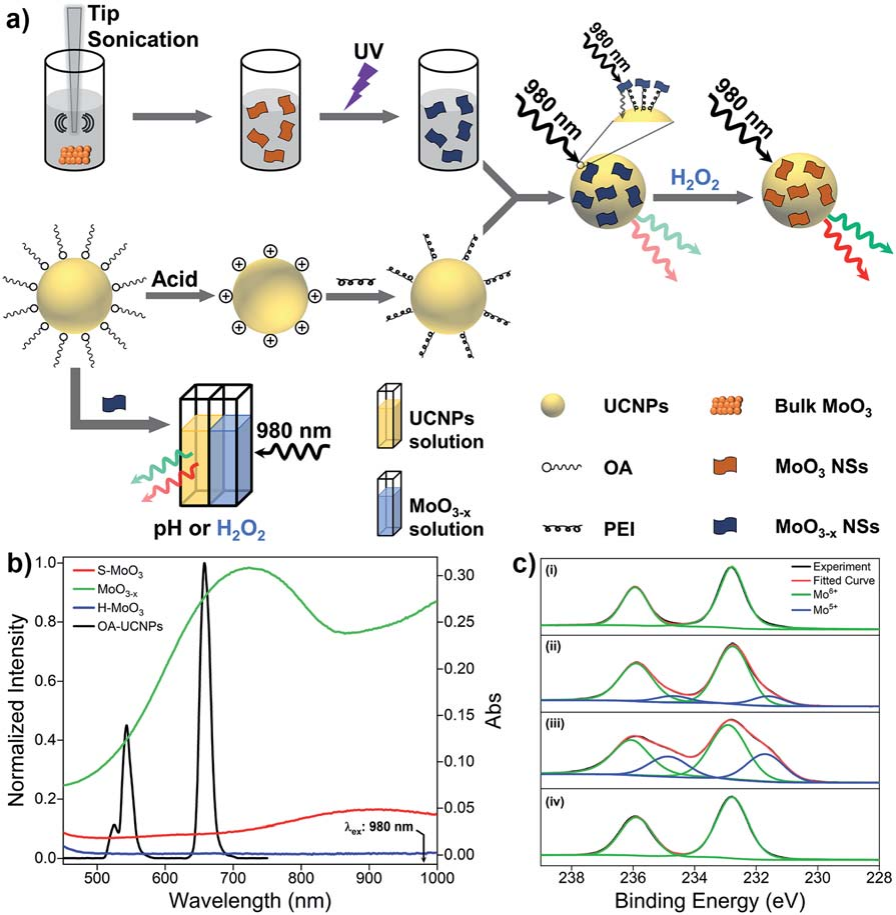
(a) Schematic illustration of the design principle of upconversion-based nanosystems for the sensing of pH and H_2_O_2_. (b) UCL spectrum of OA-UCNPs under 980 nm excitation and UV-vis spectra of S-MoO_3_, MoO_3−*x*_, and H-MoO_3_ NSs with concentration of 1 mg mL^−1^. (c) The Mo 3d XPS spectra of (i) pristine MoO_3_, (ii) S-MoO_3_ NSs, (iii) MoO_3−*x*_ NSs, and (iv) H-MoO_3_ NSs.

The design strategy of UCNPs/MoO_3−*x*_ nanocomposites for fluorescence sensing of H_2_O_2_ is based on the modulation of MoO_3−*x*_ NSs-induced reduction in UCL emissions by H_2_O_2_ through the joint effect of EEA and FRET. In contrast, the pH and H_2_O_2_ dual-responsive upconversion-based nanosystem is realized by the direct adjustment of the excitation energy for UCNPs in the non-contact mode (Fig. 1a).

Without modifications, UCNPs give rise to green and red luminescence emissions under 980 nm excitation. After the reduction of MoO_3_ by UV light, the oxygen-deficient MoO_3−*x*_ NSs exhibit strong absorption in both visible and NIR regions, overlapping well with the UCL emissions of UCNPs and the excitation wavelength for UCNPs of 980 nm (Fig. 1b). Owing to the strong NIR absorption of MoO_3−*x*_ NSs attached on UCNPs, the EEA will first take place in the UCNPs/MoO_3−*x*_ system when activated by the 980 nm light, resulting in a lowered intensity of excitation light arriving at the UCNPs, thus weakening the resulting luminescence emissions. Moreover, the efficient FRET process occurs through the spectral overlap between the absorption of MoO_3−*x*_ NSs and the UCL of UCNPs in the visible region, leading to a further decrease in the intensity of luminescence emissions. Thus, the quenching in UCL of UCNPs is efficiently achieved by the joint effect of the EEA and FRET. However, upon the addition of H_2_O_2_, the oxygen-deficient MoO_3−*x*_ NSs can be oxidized back to MoO_3_ (denoted as H-MoO_3_), leading to the decrease of absorption in the visible and NIR regions (Fig. 1b), resulting in the recovery of UCL emissions *via* the reduction in EEA and FRET. Additionally, XPS was performed to evaluate the valence state of Mo in these nanosheets. As shown in Fig. 1c, the doublet peaks (235.9 eV and 232.8 eV) in the pristine MoO_3_ sample are assigned to the binding energies of the 3d_3/2_ and 3d_5/2_ orbital electrons of Mo^6+^. After treatment by tip-sonication, two new peaks at lower binding energies (234.7 eV and 231.6 eV) appear in the obtained S-MoO_3_ NSs, which can be assigned to the Mo^5+^ oxidation state, and the integral area ratio of Mo^5+^/Mo^6+^ is calculated to be 17.1% from the XPS spectrum. This phenomenon indicates that the MoO_3_ is slightly reduced during the exfoliation process, showing weak absorption ability of S-MoO_3_ NSs in visible and NIR regions (Fig. 1b). Furthermore, the peak area ratio of Mo^5+^/Mo^6+^ increases to 47.9%, suggesting that oxygen-deficient MoO_3−*x*_ NSs are formed, where one-third of the Mo^6+^ is reduced upon UV irritation. However, the peaks at lower binding energies disappear after the addition of H_2_O_2_, confirming that MoO_3−*x*_ NSs have been oxidized. Thus, H_2_O_2_-involved oxidation of MoO_3−*x*_ enables the ability of UCNPs/MoO_3−*x*_ nanoprobes for H_2_O_2_ sensing with high sensitivity. Additionally, the adjustment of pH or addition of H_2_O_2_ in the acidic environment will lead to the variation of MoO_3−*x*_ NSs in NIR absorption, and thus fluorescence sensing of pH and H_2_O_2_ can be achieved through the direct modulation of MoO_3−*x*_ absorption-induced EEA in the non-contact mode.

### Characterization of UCNPs, MoO_3−*x*_ NSs, and UCNPs/MoO_3−*x*_ nanocomposites

**Fig. 2:**
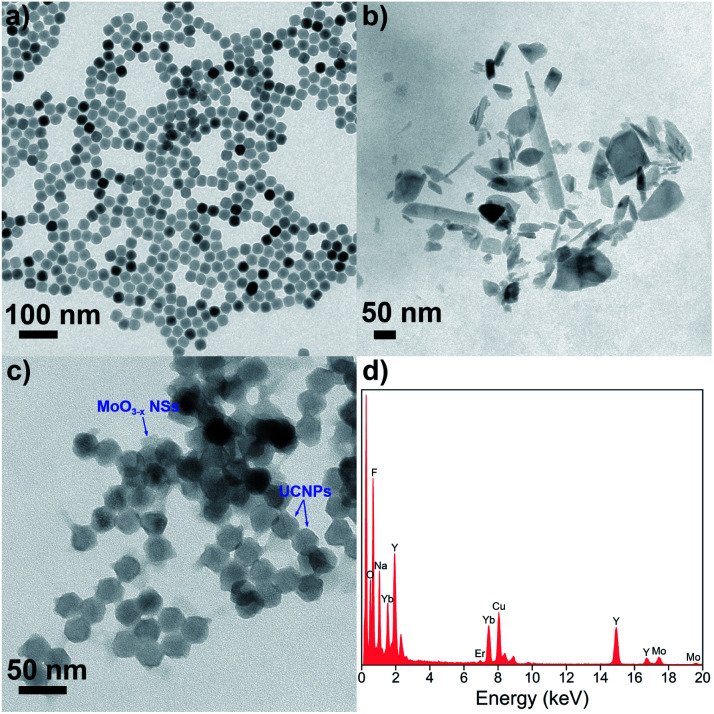
TEM images of (a) OA-UCNPs, (b) MoO_3−*x*_ NSs and (c) UCNPs/MoO_3−*x*_ nanocomposites. (d) EDS spectrum of UCNPs/MoO_3−*x*_ nanocomposites.

Hydrophobic OA-UCNPs are synthesized by employing OA as the ligand *via* the high-temperature coprecipitation method.^46^ OA-UCNPs present uniform hexagonal shape with a mean diameter of about 28 nm, which is revealed by the TEM measurement (Fig. 2a). The XRD pattern of the obtained OA-UCNPs with well-defined diffraction peaks agrees well with the standard data of hexagonal-phase NaYF_4_ (JCPDS no. 28-1192), demonstrating their high crystallinity (Fig. S1†). Ligand-free UCNPs are prepared by direct addition of formic acid to the cyclohexane solution containing OA-UCNPs through the vortexing method and sequential modification with PEI to obtain PEI-UCNPs.^47^ TEM images demonstrate unchanged morphology and size after ligand removal and polymer functionalization (Fig. S2†). The transition of OA-UCNPs to ligand-free UNCPs and further to PEI-UCNPs are confirmed by FT-IR. As shown in Fig. S3,† the transmission bands at 2926 and 2852 cm^−1^ can be assigned to asymmetric and symmetric methylene (–CH_2_–) stretching, and those at 1561 and 1460 cm^−1^ can be attributed to the vibrations of the carboxylate groups, indicating the presence of oleate ligand on the surface of OA-UCNPs. However, the disappearance of these characteristic peaks confirms the removal of surface ligand after treatment by formic acid. When further modified by PEI, new peaks appear at 3396 cm^−1^ (N–H stretching), 2930 and 2854 cm^−1^ (asymmetric and symmetric –CH_2_− stretching), and 1545 cm^−1^ (N–H bending). Accordingly, the FT-IR results verify the success in ligand removal of OA-UCNPs and further attachment of PEI on bare UCNPs. After ligand exfoliation and polymer modification, ligand-free UCNPs and PEI-UCNPs are easily dispersed in water, and the *ζ*-potentials are measured to be +35.7 mV and +32.8 mV, respectively (Fig. S4†), indicating the formation of stable colloidal solutions.

To prepare UCNPs/MoO_3−*x*_ nanoassemblies, MoO_3_ NSs are firstly prepared by tip sonication of bulk MoO_3_, and oxygen-deficient MoO_3−*x*_ NSs are easily obtained by UV irritation.^45^ As shown in Fig. 2b, the nanostructure of the MoO_3−*x*_ sample is comprised of NSs with lateral diameters in the range of 20–300 nm. UCNPs/MoO_3−*x*_ nanoassemblies are then constructed by assembling the positive charged PEI-UCNPs and negatively charged MoO_3−*x*_ NSs (Fig. S4†) *via* electrostatic interactions, as characterized by TEM (Fig. 2c). Furthermore, the EDS spectrum of UCNPs/MoO_3−*x*_ nanocomposites implies the presence of Na, F, Y, Yb, Er, Mo, and O. These results prove the successful assembling of UCNPs and MoO_3−*x*_ NSs (Fig. 2d).

Next, the optical properties of UCNPs and MoO_3−*x*_ NSs are investigated. OA-UCNPs generate green (524 and 543 nm) and red (658 nm) luminescence emissions originating from the ^2^H_11/2_ → ^4^I_15/2_, ^4^S_3/2_ → ^4^I_15/2_, and ^4^F_9/2_ → ^4^I_15/2_ transitions of Er^3+^ ions when activated by a 980 nm CW laser. The UV-vis spectroscopy of MoO_3_ NSs shows only slight absorption in visible and NIR regions. In contrast, MoO_3−*x*_ NSs strongly absorb in both visible and NIR regions, ascribed to the enhancement of the free electron concentration and the increased oxygen vacancies in the MoO_3−*x*_ NSs after exposure to UV light. The absorption of MoO_3−*x*_ NSs overlaps well with not only UCL emissions of UCNPs but also the excitation wavelength for UCNPs, namely 980 nm. Additionally, the absorption in the visible and NIR regions disappears after the addition of H_2_O_2_, as shown in Fig. 1b. The loss in the absorption intensity is due to the oxidative effect of H_2_O_2_ in the acidic medium, filling up the oxygen vacancies of MoO_3−*x*_ NSs.^48^

### Non-contact fluorescence sensing of pH

**Fig. 3:**
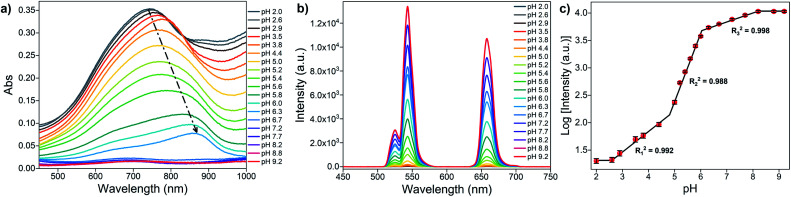
(a) UV-vis absorption spectra of MoO_3−*x*_ NSs solution (1 mg mL^−1^) at different pH values. (b) UCL spectra of OA-UCNPs in the presence of MoO_3−*x*_ NSs solutions with different pH in the non-contact mode under 4 W 980 nm excitation. (c) Relationship between the logarithm of luminescence intensity of OA-UCNPs at 658 nm and pH. Error bars represent the standard deviations of three independent measurements.

The optical properties of MoO_3−*x*_ NSs solutions (1 mg mL^−1^) at different pH are first investigated by UV-vis spectroscopy. As represented in Fig. 3a, the absorption intensity in the visible and NIR regions becomes weakened with increasing pH, and the maximum of the absorption peak gradually redshifts from 744 to 866 nm. However, no absorption peak is found in the visible and NIR region above pH 7. Moreover, the absorption at 980 nm shows the same trend as well (Fig. S5a†). This phenomenon arises from the reduction of Mo in the reduced state (returning to the Mo^VI^ state) by the addition of OH^−^ to the MoO_3−*x*_ NSs solution, leading to the reduction of free carrier concentration, and thus reducing the absorption in visible and NIR regions.^48,49^

Next, the luminescence properties are investigated by placing MoO_3−*x*_ NSs solutions (1 mg mL^−1^) with different pH in front of the OA-UCNPs solution (1 mg mL^−1^) and illuminate it then with the light of 980 nm wavelength at RT, where the 980 nm light first passes through the MoO_3−*x*_ NSs solution and then reaches OA-UCNPs (Fig. 1a). The luminescence intensity rises generally with increasing pH and remains constant above pH 8.2, as is presented in Fig. 3b. The luminescence intensity at 658 nm grows slowly when pH < 4.4, then increases remarkably in the range of 5.0 to 8.2, and the UCL shows no significant change afterward. However, the UCL intensity at 658 nm shows a nonlinear relationship with the pH, which is different from typical upconversion sensors based on the FRET process.^32–34^ Notably, we find that the logarithm of luminescence intensity at 658 nm exhibits three-separate linear regions with pH, and the linear correlation coefficient of each calibration curve is calculated to be 0.992 (pH 2.6–4.4), 0.988 (pH 5–6), and 0.998 (6.3–8.2), respectively (Fig. 3c). Thus, this upconversion-based sensor shows broad pH responsiveness in the range of 2.6 to 8.2. To investigate the reversibility of this pH sensor, the pH value of MoO_3−*x*_ NSs was adjusted from 8.2 to 2.6 and back to 8.2 by NaOH and HCl solutions for 5 cycles. As shown in Fig. S6,† the fluorescence intensity shows good reversibility of the two-way switching processes after the second cycle of pH adjustment. A slight increase in the fluorescence intensity at pH 2.6 was noticed after the first pH adjustment from 8.2, which may result from a lower reduction degree of Mo(vi) in the acidic environment than under exposure to the UV light.

### Non-contact fluorescence sensing of H_2_O_2_

**Fig. 4:**
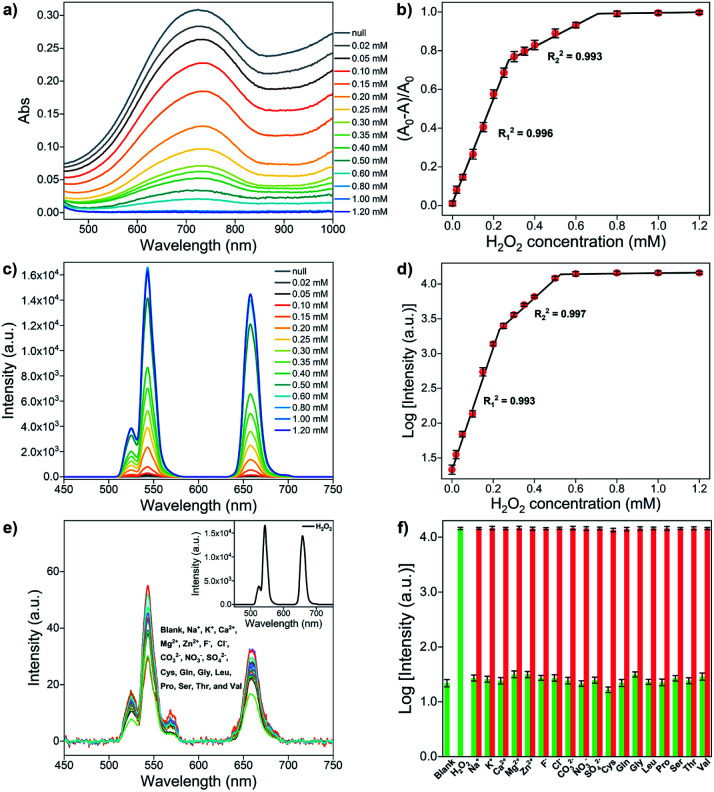
(a) UV-vis spectra of MoO_3−*x*_ NSs (1 mg mL^−1^) upon addition of different H_2_O_2_ concentrations. (b) Relationship between the change in absorbance of MoO_3−*x*_ NSs at 722 nm and H_2_O_2_ concentration. (c) UCL spectra of OA-UCNPs (1 mg mL^−1^) in the presence of MoO_3−*x*_ NSs solutions containing different H_2_O_2_ concentrations at pH 4.5 under 4 W 980 nm excitation. (d) Relationship between the logarithm of luminescence intensity of OA-UCNPs at 658 nm and the H_2_O_2_ concentration. (e) UCL spectra of OA-UCNPs in the presence of MoO_3−*x*_ NSs solutions containing 3 mM various interfering species at pH 4.5 under 4 W 980 nm excitation. Inset: UCL spectrum of OA-UCNPs in the presence of MoO_3−*x*_ NSs solutions containing 0.6 mM H_2_O_2_ at pH 4.5. (f) Changes in the logarithm of luminescence intensity of OA-UCNPs at 658 nm upon addition of 0.6 mM H_2_O_2_ and 3 mM other interfering species to MoO_3−*x*_ NSs solution at pH 4.5. Green bars represent changes in the logarithm of luminescence intensity at 658 nm upon addition of various species in MoO_3−*x*_ NSs solution, red bars represent the subsequent addition of 0.6 mM H_2_O_2_ to the above MoO_3−*x*_ NSs solution. Error bars represent the standard deviations of three independent measurements.

The sensing ability of the upconversion-based nanosystem for H_2_O_2_ in the non-contact mode is evaluated by the UV-vis absorption and UCL spectroscopy. As can be seen in the absorption spectrum (Fig. 4a), the MoO_3−*x*_ NSs solution shows a broad absorption in both visible and NIR regions, and the overall absorption intensity of MoO_3−*x*_ NSs solution decreases with the increasing amount of H_2_O_2_, and absorbance is barely observed after the addition of 0.8 mM H_2_O_2_. Notably, the maximum absorbance of MoO_3−*x*_ NSs at 722 nm decreases substantially when a low amount of H_2_O_2_ is added (<0.3 mM). Then the absorption intensity reduces gradually and no further variation in absorption is found after the addition of 0.8 mM H_2_O_2_, indicating the completion in the conversion of MoO_3−*x*_ to MoO_3_. The change in absorption intensity at 722 nm (denoted as (*A*_0_ − *A*)/*A*_0_, where *A*_0_ and *A* refer to the MoO_3−*x*_ NSs solution in the absence and presence of H_2_O_2_, respectively) shows a linear relationship with the H_2_O_2_ concentration in two-separated regions (Fig. 4b). The linear correlation coefficients of these two calibration curves are larger than 0.99, and the limit of detection (LOD) is calculated to be 0.75 μM.

The luminescence properties are then studied using similar procedures as the above-mentioned pH sensing, except that MoO_3−*x*_ solutions (1 mg mL^−1^ in acetate buffer, pH 4.5) with different added H_2_O_2_ concentrations are placed in front of the OA-UCNPs solution. The quenching efficiency (denoted as (*F*_0_ − *F*)/*F*_0_, where *F* and *F*_0_ represent the luminescence intensity at a specific wavelength in the presence and absence of MoO_3−*x*_ NSs, respectively) at 658 nm reaches 99.8% when 1 mg mL^−1^ MoO_3−*x*_ NSs solution is aligned in front of 1 mg mL^−1^ OA-UCNPs solution. When H_2_O_2_ is added in the range from 0 to 0.8 mM, the absorption intensity of MoO_3−*x*_ NSs solution at 980 nm shows a continuous decrease (Fig. S5b†). As a result, the UCL intensity of OA-UCNPs experiences a gradual uptrend in both red and green regions upon 980 nm excitation with the increasing addition of H_2_O_2_ (Fig. 4c). This can be ascribed to the oxidation of MoO_3−*x*_ to MoO_3_ by H_2_O_2_, leading to the reduction in excitation energy depletion by MoO_3−*x*_ NSs at 980 nm, and resulting in more excitation energy reached by OA-UCNPs. Similarly, like the above-discussed pH sensing in non-contact mode, the fluorescent intensity exhibits a nonlinear relationship with the H_2_O_2_ concentration as well. In addition, the logarithm of luminescence intensity at 658 nm is linearly correlated with the H_2_O_2_ concentration in the range of 0–200 μM (*R*_1_^2^ = 0.993) and 250–500 μM (*R*_2_^2^ = 0.997), respectively (Fig. 4d). According to the 3*σ* rule, the detection of H_2_O_2_ can be down to 0.63 μM, providing a lower detection limit than those reported by other upconversion-based nanoprobes (Table 1).

**Table 1:** Comparison of various upconversion-based nanoprobes for H_2_O_2_ sensing

Sensors	Mechanisms	LOD (μM)	Ref.
Benzopyrylium–coumarin-functionalized UCNPs	FRET	4.37	14
DNA-Ag/UCNPs nanocomposites	FRET	1.08	17
MnO_2_-nanosheets-modified UCNPs	FRET	0.9	26
Squaric acid-Fe(iii) & UCNPs	Inner filter effect	2.3	50
UCNPs-PDA nanosystem	FRET	0.75	51
UCNPs & MoO_3−*x*_ (non-contact mode)	EEA	0.63	This work
UCNPs/MoO_3−*x*_ nanoassemblies	EEA & FRET	9.61	This work

To further estimate the selectivity for H_2_O_2_ in the non-contact mode, the fluorescence responses of the nanosystem toward various interfering species including cations, anions, and amino acids were investigated. As shown in Fig. 4e, only the addition of H_2_O_2_ results in the recovery of the UCL emission, whereas no obvious change in luminescence intensity is observed after the addition of large excesses of the other interfering species, such as Na^+^, K^+^, Ca^2+^, Mg^2+^, Zn^2+^, F^−^, Cl^−^, CO_3_^2−^, NO_3_^−^, SO_4_^2−^, cysteine (Cys), glutamine (Gln), glycine (Gly), leucine (Leu), proline (Pro), serine (Ser), threonine (Thr), and valine (Val). Furthermore, competition experiments exhibit the recovery in UCL intensities at 658 nm, performed by adding H_2_O_2_ to MoO_3−*x*_ NSs solutions containing other interfering species (Fig. 4f). The results indicate that the sensing of H_2_O_2_ is barely affected by these coexistent species. Therefore, this system can serve as an upconversion fluorescence nanoprobe for H_2_O_2_ with high selectivity in the non-contact mode.

### Application in real sample analysis

For a practical application of the non-contact upconversion-based sensor, we studied the detection of H_2_O_2_ residue in contact lens solution, as H_2_O_2_ is usually applied in the contact lens disinfection processes and is harmful to human eyes. The results are summarized in Table 2. The recoveries of H_2_O_2_ in contact lens solutions range from 96.56% to 102.04% and the relative standard deviation (RSD, *n* = 3) values are lower than 4.45%, suggesting the efficient practical applicability of the proposed sensor.

**Table 2:** Detection of H_2_O_2_ in contact lens solution^a^

Contact lens solution	Detected (μM)	Added (μM)	Found (μM)	Recovery (%)	RSD (%)
1	ND	50	48.64	97.28	1.47
2	ND	100	102.04	102.04	4.45
3	ND	200	193.11	96.56	3.32

a ND = no detection.

### Conventional fluorescence sensing of H_2_O_2_ by UCNPs/MoO_3−*x*_ nanocomposites

**Fig. 5:**
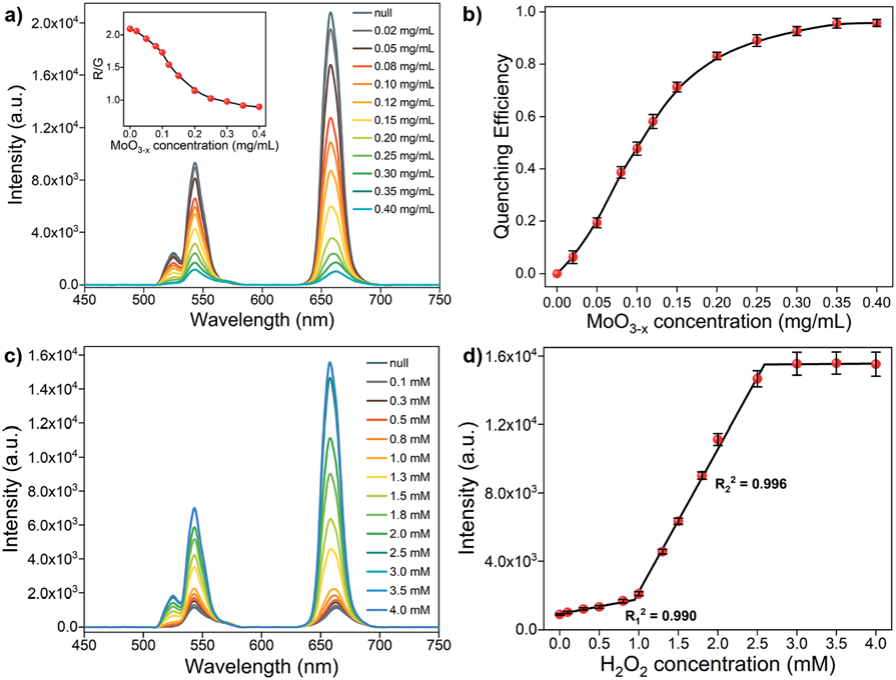
(a) UCL spectra of 0.5 mg mL^−1^ UCNPs upon the addition of different contents of MoO_3−*x*_ NSs at pH 4.5 under 4 W 980 nm excitation. Inset: R/G values of UCNPs/MoO_3−*x*_ nanocomposites with different MoO_3−*x*_ NSs contents. The black line in the inset serves as a guide to the eye. (b) Fluorescence quenching efficiency of UCNPs/MoO_3−*x*_ nanocomposites at 658 nm upon the addition of different MoO_3−*x*_ NSs concentrations at pH 4.5. The black line serves as a guide to the eye (c) UCL spectra of 0.5 mg mL^−1^ UCNPs/MoO_3−*x*_ nanocomposites (0.35 mg mL^−1^ MoO_3−*x*_ NSs) upon the addition of different H_2_O_2_ concentrations at pH 4.5 under 4 W 980 nm excitation. (d) Relationship between the fluorescence intensity of UCNPs/MoO_3−*x*_ nanocomposites at 658 nm and the H_2_O_2_ concentration. Error bars represent the standard deviations of three independent measurements.

To quantitatively analyze the quenching ability of MoO_3−*x*_ NSs on PEI-UCNPs, a series of MoO_3−*x*_ NSs modified PEI-UCNPs nanocomposites (the concentration of PEI-UCNPs is fixed at 0.5 mg mL^−1^) is prepared by changing the MoO_3−*x*_ NSs content (from 0 to 0.4 mg mL^−1^). The overlap integral (*J*(*λ*)) between the normalized emission spectrum of the donor (UCNPs) and the absorption spectrum of the acceptor (MoO_3−*x*_ NSs) is defined by the equation as follows:
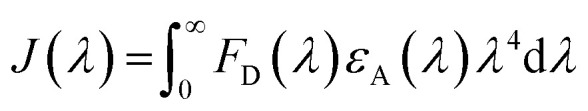
where *λ* is the wavelength in nm, *F*_D_ is the 980 nm laser-activated UCL spectrum of PEI-UCNPs normalized to an area of 1, *ε*_A_ is the extinction coefficient spectrum of MoO_3−*x*_ NSs in units of M^−1^ cm^−1^. The *J*(*λ*) value for the donor acceptor pair is calculated to be 2.79 × 10^13^ M^−1^ cm^−1^ nm^4^. The effect of different MoO_3−*x*_ NSs loading on PEI-UCNPs is evaluated by UCL spectra. As shown in Fig. 5a, the red emission intensity of UCNPs/MoO_3−*x*_ nanoassemblies experiences a significant decrease with the increasing addition of MoO_3−*x*_ NSs. Additionally, the green emission intensity of upconversion-based nanoassemblies with different loading of MoO_3−*x*_ NSs shows a similar tendency, but with a slower downward trend. As shown in Fig. 5b, the quenching efficiency at 658 nm enhances rapidly with increasing addition of MoO_3−*x*_ NSs, and shows no obvious changes after the addition of 0.35 mg mL^−1^ MoO_3−*x*_ NSs solution. Compared with unmodified PEI-UCNPs, the highest fluorescence quenching efficiency at 658 nm reaches 95.6% with the addition of 0.35 mg mL^−1^ MoO_3−*x*_ NSs. Moreover, the quenching efficiency at 543 nm shows the same trend, but with a maximum value of 87.3%. Correspondingly, UCNPs/MoO_3−*x*_ nanocomposites with the addition of 0.35 mg mL^−1^ MoO_3−*x*_ NSs are selected for the subsequent sensing experiments.

To elucidate the effect of EEA-induced reduction in the UCL of UCNPs by MoO_3−*x*_ NSs, a non-contact mode is designed. The MoO_3−*x*_ NSs solution (0.35 mg mL^−1^) is sealed in the quartz cuvette, aligning in front of another quartz cuvette containing 0.5 mg mL^−1^ PEI-UCNPs solution, and the UCL spectra are depicted in Fig. S7.† Upon the excitation by a 980 nm CW laser, the incident light first passes through the MoO_3−*x*_ NSs solution, and the energy-reduced light then reaches the UCNPs, resulting in the loss of intensity in UCL emissions. Ideally, the UCL intensity at 658 nm reduces by 72.3% compared with the control experiment.

Notably, the EEA effect will affect the intensity in all emissions, and the red-to-green emission ratio (R/G, where the red emission is integrated from 600–700 nm and the green emission is integrated from 500–600 nm) keeps its stability, which is confirmed by the activation of PEI-UCNPs (0.5 mg mL^−1^) with different power of the 980 nm laser. As presented in Fig. S8,† the red and green emission intensities increase with increasing laser power, and the R/G remains stable. However, a gradual decrease in the R/G values is observed for the UCNPs/MoO_3−*x*_ nanocomposites with the increasing loading content of MoO_3−*x*_ NSs (inset of Fig. 5a). This phenomenon can be attributed to the FRET-induced fluorescence quenching by MoO_3−*x*_ NSs, where the quenching ability by MoO_3−*x*_ NSs in red emission is more pronounced than in the green region. As discussed above, the fluorescence quenching of UCNPs by MoO_3−*x*_ NSs is achieved by the joint effect of EEA and FRET, owing to the strong absorbance ability of MoO_3−*x*_ NSs in both visible and NIR regions.

The sensing performance of UCNPs/MoO_3−*x*_ nanoassemblies toward H_2_O_2_ is investigated by UCL emission spectroscopy. As shown in Fig. 5c, the UCL emission intensity in red and green regions increases with the increasing addition of H_2_O_2_ solution. As discussed above, the addition of H_2_O_2_ leads to the oxidation of MoO_3−*x*_, resulting in the reduction in the absorption in both visible and NIR regions, and thus inhibiting the EEA effect at 980 nm and FRET process from the UCL of UCNPs to absorption of MoO_3−*x*_ in the visible region, corresponding to the enhancement of UCL emission intensity. However, the fluorescence intensity shows no obvious changes if more than 3.0 mM H_2_O_2_ are added. The fluorescence intensity at 658 nm exhibits a linear correlation to the H_2_O_2_ concentration in the range of 0–0.8 mM (*R*_1_^2^ = 0.990) and 1.0–2.5 mM (*R*_2_^2^ = 0.996), respectively (Fig. 5d). The detection limit of H_2_O_2_ is calculated to be 9.61 μM according to the 3*σ* rule. Intriguingly, the addition of a low concentration of H_2_O_2_ only leads to slight UCL recovery, while significant UCL recovery takes place with the addition of a large amount of H_2_O_2_, showing an opposite trend when compared to the sensing of H_2_O_2_ in the non-contact mode (Fig. 4d). Moreover, much more H_2_O_2_ is required for the recovery of UCL in the conventional UCNPs/MoO_3−*x*_ system. This phenomenon may be attributed to the structure of the stacked MoO_3−*x*_ NSs on UCNPs, slowing H_2_O_2_ to fill up the oxygen vacancies in MoO_3−*x*_ NSs, and thus more H_2_O_2_ is consumed for complete oxidation of MoO_3−*x*_.

## Conclusions

In summary, we have designed two different methods (*i.e.*, a non-contact method and a conventional method) for upconversion fluorescence sensing of H_2_O_2_. The non-contact method relies on the MoO_3−*x*_ NSs absorption-induced EEA effect and operates by placing the MoO_3−*x*_ NSs solution in front of UCNPs solution, whereas the conventional upconversion-based fluorescence nanoprobe, based on the joint effect of EEA and FRET, was constructed by the integration of UCNPs and MoO_3−*x*_ NSs *via* electrostatic interactions. An advantage of the non-contact method is that the valuable sensor particles do not become consumed or contaminated during the measurement and can be reused for a long time. The MoO_3−*x*_ NSs act as the quencher in both nanosystems, owing to the strong absorptive capacity of MoO_3−*x*_ in both visible and NIR regions. However, the addition of H_2_O_2_ leads to the oxidation of MoO_3−*x*_, resulting in the recovery of UCL emissions, and thus enabling the quantitative detection of H_2_O_2_ by both methods. Benefiting from the non-contact method, hydrophobic OA-UCNPs can be applied as the luminophore directly and ultrahigh fluorescence quenching (99.8%) is obtained. Moreover, the non-contact method exhibits high sensitivity toward H_2_O_2_ down to 0.63 μM, which is lower than that determined by the spectrophotometry of MoO_3−*x*_ (0.75 μM) and conventional UCNPs/MoO_3−*x*_ nanocomposites (9.61 μM). Additionally, pH sensing can be achieved by employing the non-contact mode as well, which has shown a broad pH-responsive range from 2.6 to 8.2. We believe that these results could provide new insights into the design of upconversion-based nanosystems for fluorescence sensing of other analytes.

## Conflicts of interest

There are no conflicts to declare.

## Supplementary Material

NA-003-D0NA01045F-s001
