# Supplementary material for: Upconversion-based nanosystems for fluorescence sensing of pH and H2O2
Source: Nanoscale Adv. 2021 Mar 18;3(9):2538–46. doi: 10.1039/d0na01045f (PMC9417827; doi:10.1039/d0na01045f)
Supplement: NA-003-D0NA01045F-s001 [file NA-003-D0NA01045F-s001.pdf]

Electronic Supplementary Material (ESI) for Nanoscale Advances.
This journal is © The Royal Society of Chemistry 2021

Supplementary Information

Upconversion-based nanosystems for fluorescence sensing of pH and H_2O_2

Chunning Sun* and Michael Gradzielski*

Stranski-Laboratorium für Physikalische und Theoretische Chemie, Institut für Chemie, Technische Universität Berlin, Strasse des 17. Juni 124, 10623 Berlin, Germany

*Corresponding authors:

E-mail: chunning.sun@campus.tu-berlin.de (C. Sun), michael.gradzielski@tu-berlin.de (M. Gradzielski)

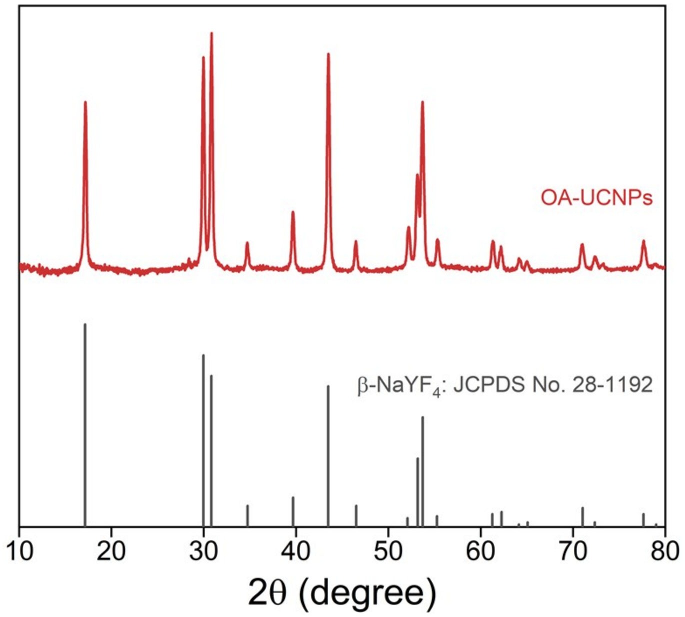

Fig. S1 XRD patterns of OA-UCNPs and the standard data of hexagonal $NaYF_4$ (JCPDS No. 28-1192).

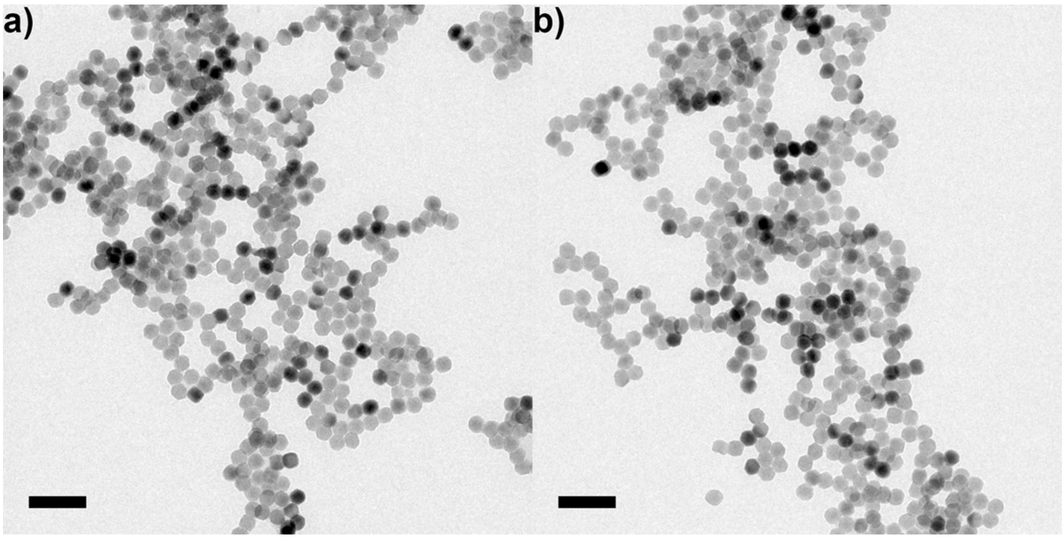

Fig. S2 TEM images of (a) ligand-free UCNPs and (b) PEI-UCNPs. Scale bars: 100 nm.

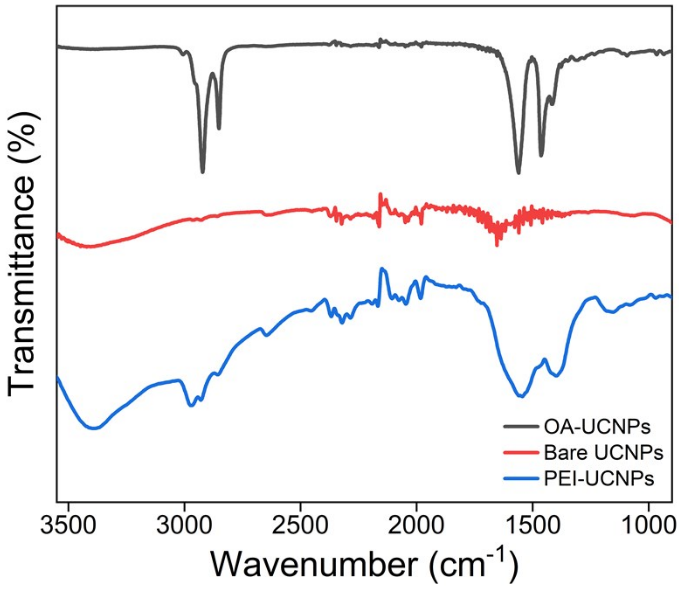

Fig. S3 FT-IR spectra of OA-, ligand-free, and PEI-UCNPs.

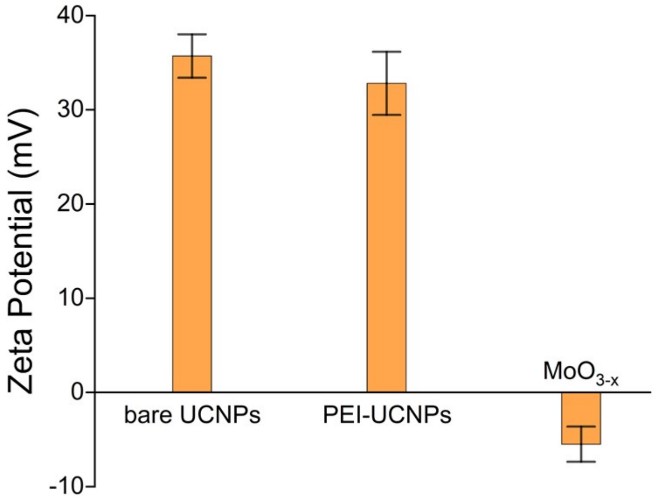

Fig. S4 Zeta potential of bare UCNPs, PEI-UCNPs, and MoO_{3-x} NSs at pH 4.5. Error bars represent the standard deviations of three independent measurements.

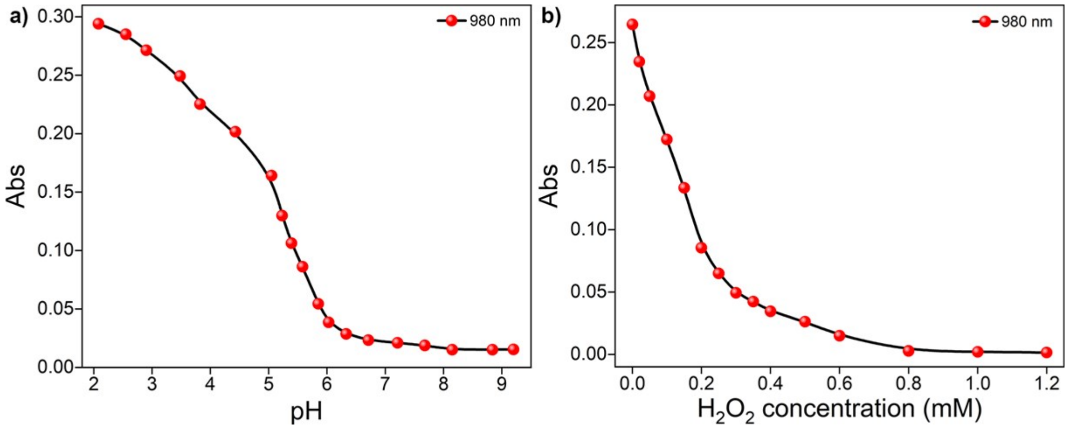

Fig. S5 (a) The absorbance of MoO_{3-x} NSs solution at 980 nm as a function of pH. (b) Absorption intensities of MoO_{3-x} NSs solution at 980 nm with the addition of different H_2O_2 concentrations at pH 4.5. Black lines serve as a guide to the eye.

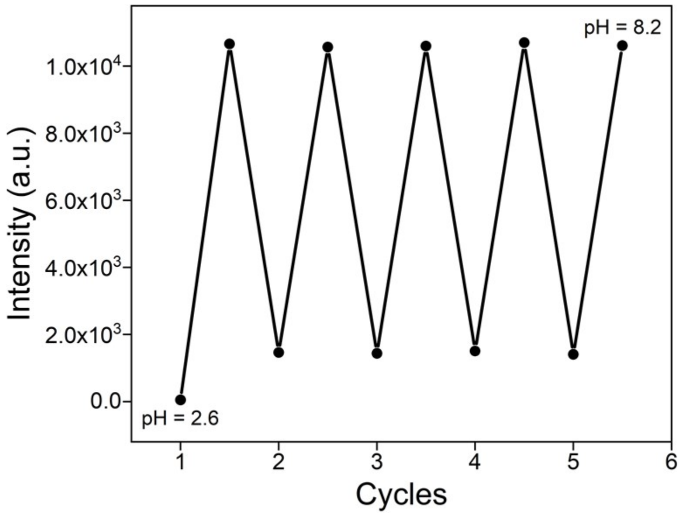

Fig. S6 Fluorescence intensity of OA-UCNPs at 658 nm in the presence of MoO_{3-x} NSs solution by altering the pH value in the non-contact mode.

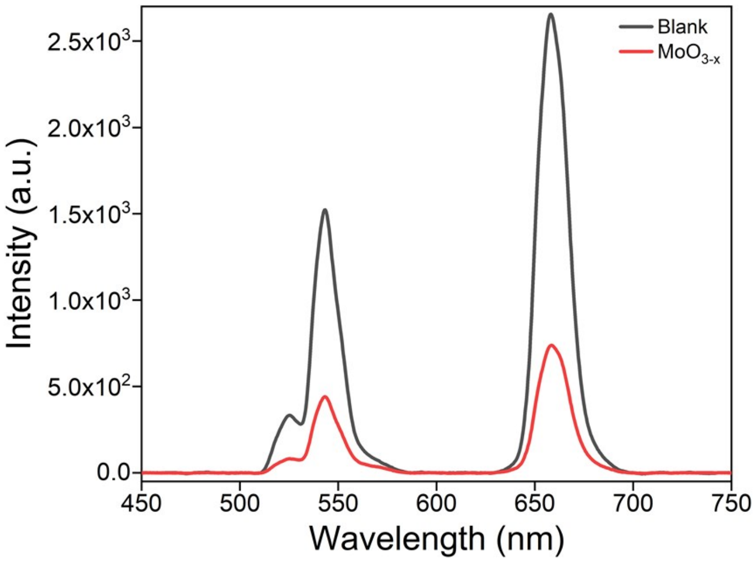

Fig. S7 UCL spectra of 0.5 mg/mL PEI-UCNPs in the absence and presence of MoO_{3-x} NSs solution (0.35 mg/mL) in non-contact mode.

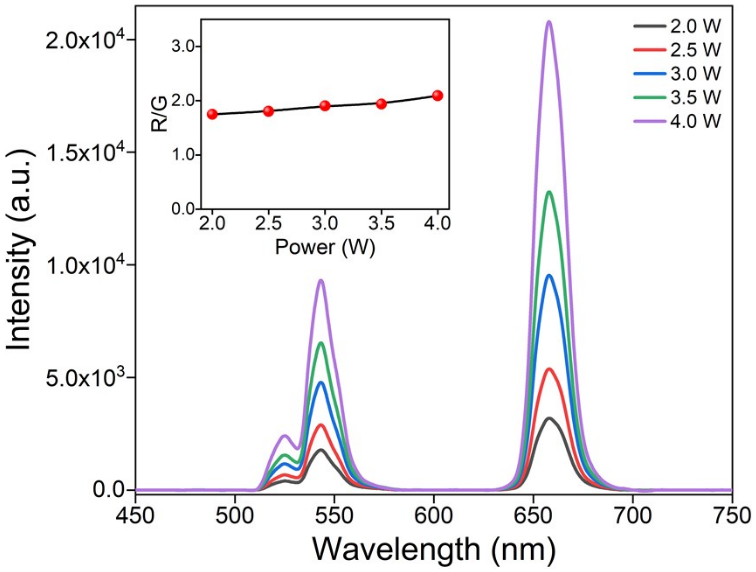

Fig. S8 UCL spectra of 0.5 mg/mL PEI-UCNPs under excitation at 980 nm with different power. Inset: the relationship between the R/G ratio and laser power.
